# A Variable Region within the Genome of *Streptococcus pneumoniae* Contributes to Strain-Strain Variation in Virulence

**DOI:** 10.1371/journal.pone.0019650

**Published:** 2011-05-05

**Authors:** Richard M. Harvey, Uwe H. Stroeher, Abiodun D. Ogunniyi, Heidi C. Smith-Vaughan, Amanda J. Leach, James C. Paton

**Affiliations:** 1 Research Centre for Infectious Diseases, School of Molecular and Biomedical Science, University of Adelaide, Adelaide, South Australia, Australia; 2 Menzies School of Health Research, Charles Darwin University, Darwin, Northern Territory, Australia; Institut de Pharmacologie et de Biologie Structurale, France

## Abstract

The bacterial factors responsible for the variation in invasive potential between different clones and serotypes of *Streptococcus pneumoniae* are largely unknown. Therefore, the isolation of rare serotype 1 carriage strains in Indigenous Australian communities provided a unique opportunity to compare the genomes of non-invasive and invasive isolates of the same serotype in order to identify such factors. The human virulence status of non-invasive, intermediately virulent and highly virulent serotype 1 isolates was reflected in mice and showed that whilst both human non-invasive and highly virulent isolates were able to colonize the murine nasopharynx equally, only the human highly virulent isolates were able to invade and survive in the murine lungs and blood. Genomic sequencing comparisons between these isolates identified 8 regions >1 kb in size that were specific to only the highly virulent isolates, and included a version of the pneumococcal pathogenicity island 1 variable region (PPI-1v), phage-associated adherence factors, transporters and metabolic enzymes. In particular, a phage-associated endolysin, a putative iron/lead permease and an operon within PPI-1v exhibited niche-specific changes in expression that suggest important roles for these genes in the lungs and blood. Moreover, *in vivo* competition between pneumococci carrying PPI-1v derivatives representing the two identified versions of the region showed that the version of PPI-1v in the highly virulent isolates was more competitive than the version from the less virulent isolates in the nasopharyngeal tissue, blood and lungs. This study is the first to perform genomic comparisons between serotype 1 isolates with distinct virulence profiles that correlate between mice and humans, and has highlighted the important role that hypervariable genomic loci, such as PPI-1v, play in pneumococcal disease. The findings of this study have important implications for understanding the processes that drive progression from colonization to invasive disease and will help direct the development of novel therapeutic strategies.

## Introduction

*Streptococcus pneumoniae* (the pneumococcus) is a leading cause of bacterial pneumonia, invasive disease (bacteremia and meningitis [IPD]) and otitis media, and is responsible for >1 million deaths in children <5 years of age annually [1]. However, the ability of different serotypes and clones to cause IPD varies and has led to the grouping of serotypes and clonal clusters according to invasive potential [2]–[5]. In particular, serotype 1 pneumococci have repeatedly been reported to have a high invasive potential due to the rarity of asymptomatic carriage [2], [5]. Furthermore, serotype 1 isolates frequently cause disease in patients without an underlying illness and as such behave as a primary pathogen [6]. In spite of this, whilst serotype 1 isolates have a high invasive potential, commonly studied serotype 1 clones tend to cause less severe disease in both humans and mice when compared to certain other serotypes and clones that behave as opportunistic pathogens [4], [6]. However, a number of clones from the ST217 clonal cluster (CC217) have been responsible for African epidemics of IPD with unusually high mortality rates, and are considered to be hypervirulent. In contrast to these hypervirulent clones, relatively high rates of serotype 1 asymptomatic carriage have been reported in a number of communities following vaccination with the 7-valent conjugate vaccine [7], [8]. In particular, serotype 1 carriage by ST304 and ST227 clones was detected in a number of remote Indigenous communities in Australia, without an associated increase in serotype 1 IPD in the same communities [8]. Therefore, it is clear that considerable variation in virulence exists between strains of the same serotype, which in turn highlights the contribution that serotype-independent factors play in virulence. Of particular interest is the existence of many ‘accessory regions’ (AR) within the pneumococcal genome that may contribute to such differences in virulence [9]–[14]. However, whilst a number of potential virulence determinants such as the pilus encoded within the *rlrA* islet and the pneumococcal serine rich repeat protein (PsrP) have received particular attention, little consistency between the presence of specific virulence determinants and invasive potential has been found in large-scale comparisons [10], [14]–[21]. Nevertheless, it is possible that such large-scale comparisons fail to take into account the significant differences in virulence that may exist within groups of isolates with apparently equivalent invasive potential, such as hypervirulent and moderately virulent serotype 1 clones [14]. In addition, comparisons across serotypes risk underestimating the impact of the serotype itself on virulence, due to serotype-specific structural differences in the capsule that can affect complement deposition and resistance against phagocytosis [22]–[25]. Therefore, by comparing the genomes of non-invasive and invasive serotype 1 isolates, it is possible to identify serotype-independent factors that alter the outcome of infection. Initially, the human virulence status of non-invasive and invasive serotype 1 isolates was confirmed in mouse models of infection, enabling these isolates to be grouped as non-invasive, intermediately virulent and highly virulent. Using these virulence profiles as a basis, preliminary comparisons between the three virulence phenotypes were performed using comparative genomic hybridization (CGH), and next generation genome sequencing technology. These comparisons identified a number of previously described ARs as well as new ARs that were present only in the highly virulent isolates. Of particular significance was that the highly virulent isolates harbored a version of the pneumococcal pathogenicity island 1 (PPI-1) that conferred greater competitiveness *in vivo* than the versions in less virulent isolates.

## Results and Discussion

### The virulence of serotype 1 isolates in mouse models of infection mimics human disease

**Figure 1:**
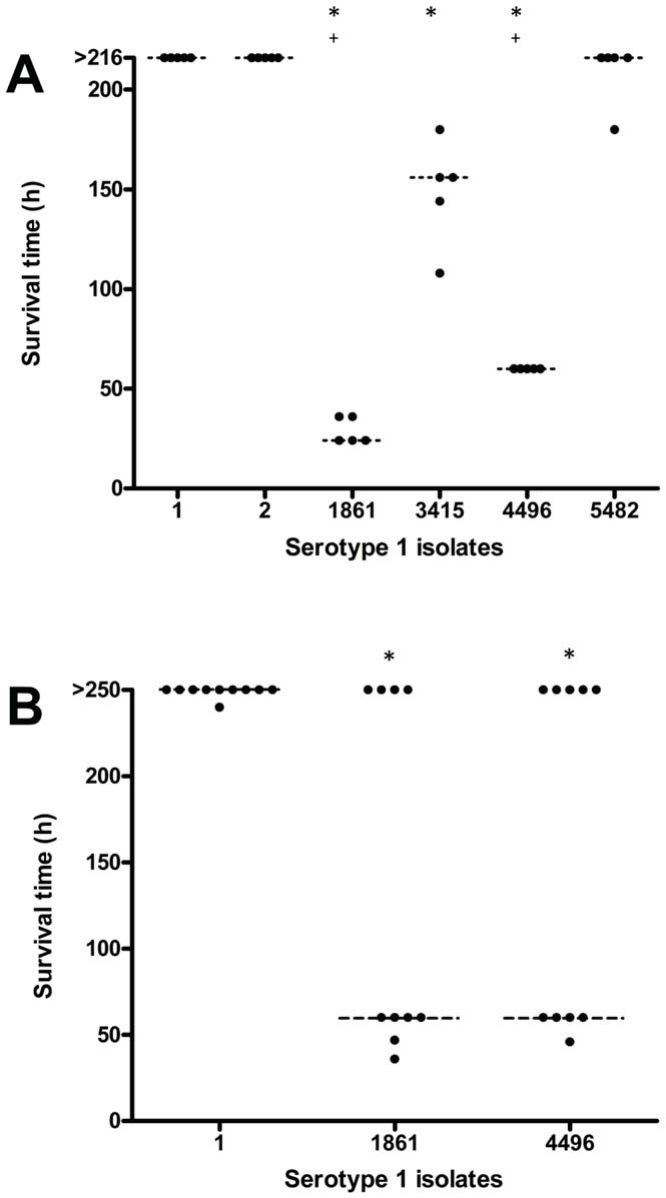
Confirmation of virulence in mice of non-invasive and invasive serotype 1 isolates. Survival times were recorded following i.p. (A) or i.n. (B) challenge with approximately 10^4^ CFU and 10^7^ CFU of the relevant strain, respectively. Experiments were undertaken for 216 h and 252 h following i.p. and i.n. challenge, respectively. Horizontal broken lines indicate the median survival time. Statistical significance was calculated using the two-tailed Mann-Whitney *U* test. (*, ^+^, *P*<0.05). ‘*’ indicates comparison with non-invasive isolates; ‘^+^’ indicates comparison with intermediately virulent isolates.

**Figure 2:**
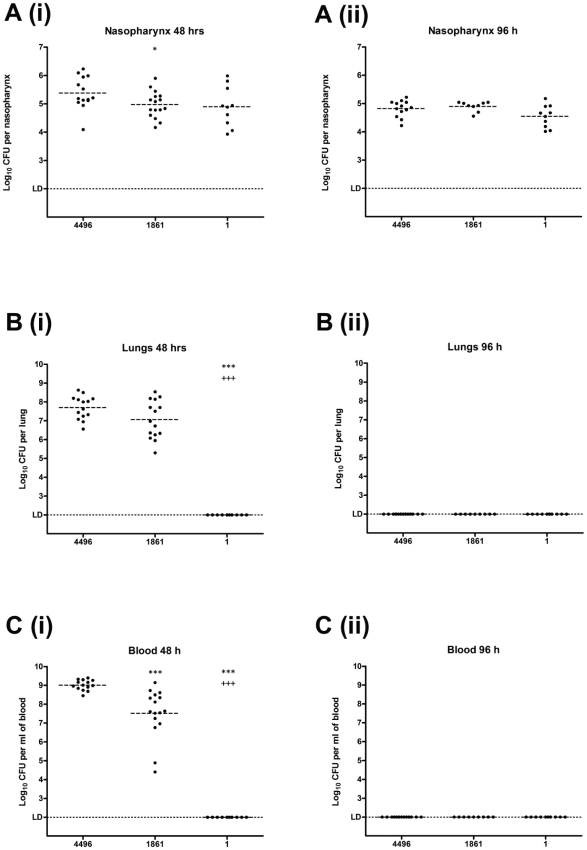
Number of *S. pneumoniae* CFU recovered from the nasopharynx, lungs and blood of infected mice. The number of CFU recovered from the nasopharynx (A), lungs (B) and blood (C) of CD1 mice was determined at 48 h (i) and 96 h (ii) post-challenge. The horizontal broken lines in each strain group indicate the geometric mean number of CFU that were recovered. Statistical differences were analyzed by two-tailed unpaired *t*-test on log-transformed values (*, *P*<0.05; **, *P*<0.01; ***, +++ *P*<0.001). ‘*’ indicates comparison with 4496 and ‘^+^’ indicates comparison with 1861. The single horizontal spotted line indicates the limit of detection (LD) which equates to 10^2^ CFU/nasopharynx, 2×10^2^ CFU/lung and 10^2^ CFU/ml blood.

An intraperitoneal (i.p.) mouse challenge model identified three distinct virulence phenotypes by comparing the virulence of a collection of serotype 1 isolates (Fig. 1A). Menzies^1^-1 (strain 1) and Menzies^1^-2 (strain 2) were avirulent in mice, which mimicked their non-invasiveness in humans. Menzies^1^-1861 (strain 1861) and WCH4496 (strain 4496) were highly virulent in mice, as mice infected with these strains had significantly reduced survival times than those challenged with the other two invasive isolates. Menzies^1^-3415 (strain 3415) and Menzies^1^-5482 (strain 5482) were considered to be intermediately virulent, as they were not carried asymptomatically in humans, but they were less virulent than strains 1861 and 4496 in mice. The virulence of strains 3415 and 5482 appears to be similar to the relatively low virulence of most serotype 1 isolates in mice that has been reported previously [4], [6]. An intranasal (i.n.) mouse challenge model also confirmed the differences in virulence between the non-invasive strain 1 and the highly virulent strains 1861 and 4496 (Fig. 1B). A feature of the highly virulent isolates was their ability to either cause rapidly fulminant infection within 60 h of challenge, or not cause detectable disease at all. Interestingly, daily analysis of bacteremia in all mice revealed that in surviving mice pneumococci did not reach a detectable level within the blood at any time (data not shown). Further characterization of the pathogenicity of strains 1, 1861 and 4496 was performed by comparing the recovery of pneumococci in the nasopharynx, blood and lungs at both 48 h and 96 h post-challenge using an i.n. mouse challenge model (Fig. 2). At 48 h a small but significant difference was observed between the number of pneumococci in the nasopharynx between strains 1861 and 4496 (Fig. 2A[i]). However, there was no significant difference in the number of bacteria recovered from the nasopharynx of strain 1-infected mice when compared to either of the highly virulent strains at either timepoint (Fig. 2A[ii]). No pneumococci were recovered from either the blood or lungs of strain 1-infected mice, which was in stark contrast to the numbers recovered from both strain 1861- and 4496-infected mice at 48 h (Fig. 2B[i] and C[i]). Whilst there was a small, but significant difference in the level of bacteremia between strains 1861 and 4496, this difference was minimal compared to strain 1. As shown in Figure 2, mice challenged with either of the highly virulent strains either develop fulminant infection within 60 h or survive the challenge completely. Therefore, the strain 1861- and 4496-infected mice analyzed at 96 h represent surviving mice, and unsurprisingly lack detectable numbers of pneumococci in either the blood or lungs (Fig. 2B[ii] and 2C[ii]). The fact that a proportion of mice completely survive i.n. challenge is most likely a consequence of mouse-mouse variation in the actual number of pneumococci aspirated into the lungs immediately following i.n. challenge. However, most importantly the key difference between the non-invasive and highly virulent phenotypes is the ability of the latter to invade and survive in the blood and lungs. We used MLST to examine whether the strains with differing virulence profiles had any clonal relationship. Strains 1 and 2 (ST304), and strains 3415 and 5482 (ST227), belong to the lineage A of serotype 1 clones [26], whereas the highly invasive strains 1861 (ST3079) and 4496 (ST3018) belong to lineages B and C, respectively. In particular, strain 1861 was found to be a single-locus variant of ST217 and a double-locus variant of ST618 and ST303, which were the dominant clones responsible for epidemics of severe serotype 1 IPD in parts of Africa [27]–[29]. Therefore, the heightened virulence of strain 1861 in mice is consistent with the severity of disease in humans caused by clonally-related strains. A summary of the virulence and MLST data of the serotype 1 isolates used in this study is shown in Table 1.

**Table 1:** Serotype 1 isolates used in this study.

Isolate	ST (Lineage*)	Virulence status+
Menzies^1^-1	304 (A)	Non-invasive
Menzies^1^-2	304 (A)	Non-invasive
Menzies^1^-3415	227 (A)	Intermediately virulent
Menzies^1^-5482	227 (A)	Intermediately virulent
Menzies^1^-1861	3079 (B)	Highly virulent
WCH4496∧	3018 (C)	Highly virulent

*Lineage is as described in Brueggemann *et al.* [26].

∧ Non-Indigenous isolate. Other isolates were all of Indigenous origin.

+ Non-invasive isolates were both non-invasive in humans and mice: intermediately virulent strains were virulent in humans, but significantly less virulent in mice than strains 1861 and 4496. Highly virulent strains were significantly more virulent than the other four strains.

### Genetic differences between the non-invasive, intermediately virulent and highly virulent strains were identified by genomic sequencing

Genome comparisons were performed between the serotype 1 isolates in order to identify ARs present in the highly virulent strains that might be responsible for their heightened virulence. Genomic comparisons were initially performed by sequencing the genomes of strains 1 and 1861. The presence of regions >1 kb in size that were present only in strain 1861 was subsequently tested in all six serotype 1 isolates by PCR to identify those regions associated with heightened virulence. The primers used in these PCRs are listed in Table S1. In this study, we focused attention on regions >1 kb, as these were likely to encode an intact gene product, and probably represent horizontally acquired genetic material. Of course, the importance of smaller regions (<1 kb), which might encode bacteriocins, signaling peptides, or regulatory RNAs, as well as single nucleotide polymorphisms (SNPs) in coding and non-coding regions should not be discounted. Nevertheless, the expansive pool of ARs available to the pneumococcal genome warranted paying particular attention to these larger regions. The presence of key regions that have previously been associated with virulence in other studies but not associated with virulence in this study was confirmed by comparative genomic hybridization (CGH). The P1031 (ST303; lineage B) and INV104B (ST227; lineage A) genome sequences were most closely aligned with strains 1861 and 1, respectively.

### Regions associated with heightened virulence

Eight regions >1 kb in size were identified in both of the highly virulent isolates but absent in all four less invasive isolates (Table 2). A detailed list of each gene present in each AR is included in Table S2. Of these regions, those designated 1, 2, 5, 6 and 7 have not previously been identified as ARs [14].

**Table 2:** Genomic regions present only in the genomes of the highly virulent isolates.

Region (AR)*	Size	Putative annotation/function of key gene(s) in region	Homologous ORFs			Other genomes#						
			P1031	TIGR4	D39	A	B	C	D	E	F	G
1	35 kb	Group 1 pneumophage (*pblB*-like, endolysin)	0028–0083	-	-	−	∧	∧	−	∧	−	−
2	1 kb	Sodium-dependent permease, transcription regulator (MerR)	0750–0751	0739–0740	0643–0644	+	+	+	+	−	+	+
3 (22)	6.3 kb	PPI-1v (PezAT, NplT)	1049–1058	1046–1056	0927–0936	+	+	+	−	−	−	+
4 (22)	8 kb	PPI-1v (Metabolic/hypothetical)	1059–1069	-	-	−	−	−	−	−	+	−
5	58 kb	ZmpD, Tn*5253* (TA system, UmuCD)	1141–1198	-	-	+	−	−	−	−	∧	∧
6	1.7 kb	High-affinity iron/lead permease	1340–1343	-	1155–1157	−	−	−	−	∧	−	+
7	2.9 kb	ABC-type transporter, transcription regulator (ArsR)	1637–1643	-	-	−	−	−	−	−	−	−
8 (28)	18 kb	ABC transporter, sialic acid degradation enzymes	1350–1371	1346–1349∧	1164–1183	∧	∧	∧	−	∧	∧	∧

*AR designated by Bloomberg *et al.* [14].

# Presence in other *S. pneumoniae* genomes; A, ATCC 700669; B, JJA; C, 70585; D, Taiwan^19F^-14 ; E, Hungary^19A^-6; F, G54; G, CGSP14.

+, Region present; ∧, Region partially present; −, Region absent; Confirmed by BLAST searches of the KEGG database.

A detailed list of genes present in each AR is included in Table S2.

Region 1 consists of a temperate bacteriophage genome with greatest homology to *SPP_0028–0084* in P1031 and to a lesser extent the *Streptococcus oralis* PH10 phage [30]. The prophage is inserted into a position between genes homologous to *SP_0019* (adenylosuccinate synthetase) and *0020* (cytidine/deoxycytidylate deaminase) in TIGR4 and *SPINV104*_*00170* and *00180* in INV104. In addition, examination of the sequence of the integrase gene (*SPP_0028*) suggests that the phage in strains 1861 and 4496 belongs to the group 1 pneumophage [31], [32]. This prophage encodes genes homologous to platelet-binding protein B (PblB) and an endolysin, both of which are required for the virulence of *Streptococcus mitis* in an animal model of infective endocarditis [33]. PblB and the endolysin have been shown to be required by *S. mitis* for adherence to human platelets [34]–[36]. However, a similar role for the products of these genes in *S. pneumoniae* has yet to be demonstrated. Interestingly, the endolysin gene shares 80% nucleotide sequence identity with the major autolysin (*N*-acetylmuramoyl-L-alinine amidase; *lytA*), which is an important pneumococcal virulence factor [37]–[40]. Of the publicly available genome sequences, only strain P1031 contains sequences homologous to the full length of region 1 represented by the prophage. However, Hungary^19A^-6, JJA, 70585, OXC141, SPN994039, SPN034183, SPN994038, and SPN034156 possess *pblB-* and endolysin-(in addition to *lytA*-) like genes at a similar genomic location.

Region 2 encodes a putative MerR family transcriptional regulator and MutT/Nudix family protein homologous to *SPP_0750* and *0751*, respectively. In addition, the gene encoding a putative sodium-dependent permease was truncated in the non-invasive and intermediately virulent strains due to the absence of region 2. However, it is not clear whether the gene is functional in the highly virulent strains due to a frameshift mutation approximately 220 bp downstream of the start codon. Region 2 is inserted between genes homologous to *SPINV104_06130* and *06140* in INV104. The genes in region 2 are also present in numerous other genomes including TIGR4 (*SP_0737*–*0740*), D39, ATCC 700669, JJA, 70585, Taiwan^19F^-14, G54, CGSP14, INV200, SPN034156, OXC141, SPN994039, SPN034183, SPN994038 and TCH8431/19A. A MerR family transcriptional regulator in *S. pneumoniae* (*SP_1856*) has been shown to be involved in nitric oxide stress and is required for full systemic virulence [41].

**Figure 3:**
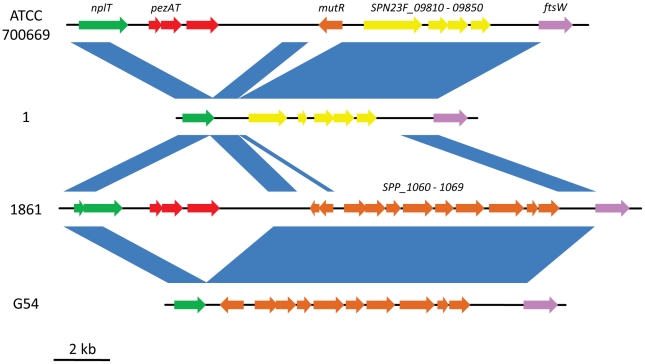
Alignment of PPI1v between ATCC 700669, 1, 1861 and G54. The nucleotide sequences of PPI-1v were aligned using the artemis comparison tool between ATCC 700669, 1, 1861 and G54. Strain 1 represents the non-invasive and intermediately virulent isolates, and strain 1861 represents the highly virulent isolates. Blue shading between strains indicates nucleotide sequence identity exceeding 90%. Green arrows indicate the *nplT* ORFs, red arrows indicate the *pezAT* operon, orange arrows indicate ORFs within divergent sequence that are absent in strain 1, yellow arrows indicate ORFs that are absent in strain 1861 and purple indicates *ftsW*. Tn*5252* ORFs are not shown.

**Figure 4:**
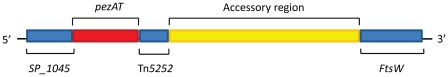
General component structure of PPI-1v. Summary of PPI-1v derived from alignments of the region between ATCC 700669, 1, 1861 and G54. Blue indicates regions of shared sequence between the aligned strains, red indicates the *pezAT* region and yellow indicates a region of divergent sequence (accessory region).

Regions 3 and 4 consist of the previously described AR 22 [14], also known as the PPI-1 variable region (PPI-1v). PPI-1v appears to be a hotspot for recombination and in some strains contains the PezAT toxin-antitoxin (TA) system, which has been implicated in virulence [42]–[44]. An alignment between PPI-1v from the lineage A isolates and the highly virulent isolates shows the relative position of regions 3 and 4 and is compared with the region in ATCC 700669 and G54 (Fig. 3). In addition, this alignment shows that PPI-1v consists of two variable components: the *pezAT* region (region 3) and an accessory region (region 4) (Fig. 4). In this study, *pezAT* was present only in the highly virulent isolates and not in either the intermediately virulent or non-invasive isolates (Table 2). In addition to *pezAT* itself, the non-invasive and intermediately virulent isolates lack most of the neopullulanase gene (*SP_1046*; *nplT*). However, since *nplT* is fragmented in strains 1861 and 4496, it is unlikely that a functional protein is produced by these strains. The *pezAT* region is present in numerous other strains including JJA, ATCC 700669, CGSP14, OXC141, SPN034156, SPN034183, SPN994038, D39, INV200 and 70585 and as such is not unique to 1861 and 4496. Between regions 3 and 4 is a 3-kb region of a Tn*5252*-like sequence that is approximately 95% identical in all six strains (Fig. 3). Within this 3-kb region is a 1.5-kb deletion in the non-invasive and intermediately virulent isolates, which has led to the loss of a putative Rgg/GadR/MutR family transcriptional regulator. However, this gene is unlikely to be functional in the highly virulent strains, due to a previously characterized frameshift mutation [45]. This mutation led to the loss of *ropB* expression in *S. pyogenes*, which was responsible for loss of SpeB expression and reduced virulence in murine systemic models of infection. As can be seen in Fig. 3 the PPI-1v accessory region, which consists of region 4, is a section of divergent sequence. In the highly virulent strains, the accessory region is 8-kb in size and includes a putative operon encoding hypothetical proteins and metabolic enzymes such as 3-hydroxyisobutyrate dehydrogenase (3HIBDH), prephenate dehydratase (PDT) and UDP-glucose 4-epimerase (GalE). Downstream of this operon is a putative biotin carboxylase and a fragmented transporter of the major facilitator superfamily. Of the publicly available genomes, G54, 11-BS70 and MLV-016 also contain this region 4 sequence. However, unlike strains 1861 and 4496, the genomes of G54, 11-BS70 and MLV-016 lack *pezAT*. The PPI-1v accessory region in the non-invasive and intermediately virulent isolates contains a fragmented lantibiotic modification and export gene, and a putative lantibiotic immunity system ABC transporter (Table 3). However, the region lacks the structural gene for mersacidin lantibiotic and lantibiotic modifying enzymes. This version of PPI-1v has previously been described in the *pezAT*-positive strain ATCC 700669 [43] and is homologous to ORFs *SPINV104_09130–09190* in the *pezAT-*negative strain INV104. This version of the region is also present in the *pezAT*-positive strains JJA, SPN033038 and SPN032672.

**Table 3:** Genomic regions of the non-invasive and intermediately virulent isolates that replaced regions associated with the highly virulent strains.

Region (AR)	Size	Putative annotation/function of key gene(s) in region	Homologous ORFs			Other genomes#						
			INV104	TIGR4	D39	A	B	C	D	E	F	G
4 (22)	5.5 kb	PPI-1v (Mersacidin immunity ABC transporter)	09130–09190	-	-	+	−	−	−	−	−	−
8 (28)	24 kb	Polysaccharide ABC transporter (LplBC-like),	11270–11410	-	-	−	−	−	−	+	−	−

# Presence in other *S. pneumoniae* genomes; A, ATCC 700669; B, JJA; C, 70585; D, Taiwan^19F^-14 ; E, Hungary^19A^-6; F, G54; G, CGSP14.

+, Region present; ∧, Region partially present; −, Region absent; Confirmed by BLAST searches of the KEGG database.

Region 5 includes the putative zinc metalloproteinase D (ZmpD) and Tn*5253*, which are homologous to *SPP_1141*–*1198* in P1031. However, this region is not present in TIGR4 or INV104 and is inserted between homologues of *SP_1154* and *1155*, and *SPINV104_09960* and *09970*, respectively. Other pneumococcal Zmps include the IgA protease and ZmpC, which function to cleave host IgA and activate human matrix metalloprotease 9, respectively, and thus contribute to virulence [46]–[52]. However, as the substrate of ZmpD is unknown, it is not clear whether this enzyme plays a role in virulence. Other strains harboring *zmpD* include ATCC 700669, JJA, G54, CGSP14 and INV200, and the gene was detected in 49% of 218 mostly invasive disease isolates in one study [53]. The large conjugative transposon Tn*5253* is a composite element consisting of regions from Tn*916* and Tn*5252* and contains genes that confer resistance to tetracycline and chloramphenicol [54], [55]. In the highly virulent serotype 1 isolates, the region encodes a putative TA system homologous to PezAT in PPI-1 and an *umuCD*-like operon. UmuCD is involved in a branch of the SOS response in *E. coli* and in *S. pneumoniae* has been shown to confer greater UV tolerance [56], [57]. Whilst a number of other strains contain distinct Tn*5252* and Tn*916* elements with homology to region 5, ATCC 700669 also harbors the compositeTn*5253* element [43].

Region 6 encodes a putative high-affinity iron/lead permease and a fragmented DyP-type peroxidase that are homologous to *SPP_1340* and *1341*, respectively in P1031. This region is inserted between homologues of *SPINV104_11210* and *11240* in INV104 and *SP_1299* and *1306* in TIGR4. In both TIGR4 and INV104 the corresponding region consists of a number of small hypothetical genes. Whilst the substrate of the permease in region 6 requires experimental confirmation, other pneumococcal transporters of metal ions such as Psa (manganese) and Pia (iron) are important in pneumococcal pathogenesis [58]–[63]. The putative metal permease is also present in the genomes of D39, Hungary^19A^-6, CGSP14, INV200 and CDC3059-06.

Region 7 encodes a putative ArsR family transcriptional regulator, and an ABC-2 type transporter including the transmembrane and ATP-binding components homologous to *SPP_1779* and *1780* in P1031, respectively. The region is absent in both TIGR4 and INV104, and if present would be inserted between *SP_1779* and *1780*, and *SPINV104_15230* and *15250*, respectively. ArsR family transcriptional regulators are often responsive to metal ions, and like MerR regulators, have been implicated in resistance against environmental stresses [64]. Examination of other pneumococcal genome sequences revealed that strains MLV-016, 11-BS70, 9-BS68, 14-BS69 and 18-BS74 also harbor this region.

Region 8 is homologous to *SPP_1350*–*1367* in P1301, which corresponds to the previously described AR 28 [14]. In particular, the AR 28 subregion RD8b1 [10], [65] encodes a putative ABC transporter for the transport of glutathione and putative *N*-acetylmannosamine-6-phosphate 2-epimerase, kelch-like protein, glycoside hydrolase family protein and *N*-acetylneuraminate lyase genes. Much of this region is also present in D39 (*SPD_1164*–*1174*). A putative ABC transporter is also encoded within the same, but divergent region in the non-invasive and intermediately virulent isolates (Table 3). The transporter appears to be similar to the LplABC polysacchaide transporter in *Bacillus subtilis* and *Agrobacterium radiobacter* [66]. Glycoside hydrolase and *N-*acetylmannosamine-6-phosphate epimerase genes present within the region in both groups of strains share 50% nucleotide sequence identity. Putative diadenosine tetraphosphate hydrolase and dihydrolipoamide dehydrogenase genes also exist in this region in the intermediately virulent and non-invasive isolates. Furthermore, the AR 28 subregion RD8b2 that is homologous to *SP_1345–1349* in TIGR4 and includes a second ABC transporter [10], [65] is present in the non-invasive and intermediately virulent isolates, but absent in the highly virulent isolates. The RD8b3 subregion that is homologous to *SP_1345–1349* and was reported to be required for wild-type virulence in TIGR4 [65], was present in all six serotype 1 isolates in this study. The overall region in the non-invasive and intermediately virulent isolates is homologous to *INV104_11270–11410* in INV104.

### Key regions found not be associated with a virulence phenotype in this study

Previous genomic comparisons have identified a number of ARs that were associated with clonal clusters with high invasive potential. While it is likely that a number of these regions are involved in virulence, it is unlikely that they are responsible for the differences in virulence that we observed between the strains in this study. ARs 5, 6, 9, 15, 20, 21, 29 and 39 were reported to be present in the majority of tested isolates from highly invasive clonal clusters [14]. In particular, ARs 5, 6, 15 and 29 have previously been shown to impact virulence using signature tagged mutagenesis (STM) [67], [68]. However, AR 5 is absent in all six isolates in this study (Table 4), and AR 6 is present only in the lineage A isolates and not in the highly virulent isolates. ARs 9, 15, 20, 21, 29 and 39 were present in all six strains, which is not surprising since Blomberg *et al.* [14] found that these regions are present in the majority of serotype 1 isolates that were tested in their study [14]. However, this finding implies that these regions are not responsible for the virulence differences between the isolates in this study. In addition, AR 6 is also not likely to be required for the heightened virulence of strains 1861 and 4496. Furthermore, ARs 10, 16, 19 and 27 were shown to be present in some serotype 1 isolates, and not others [14]. In this study ARs 16 and 19 were present in all isolates and AR 10 was present in the lineage A isolates, but not the highly virulent isolates (Table 4). Similar to Blomberg *et al.*, [14] the presence of AR 27 did not appear to correlate with the invasive potential of the strains in this study (Table 4) [10], [14]. Whilst the deletion of AR 31 has previously been reported to significantly reduce the virulence of TIGR4 [65], the region was present in strain 1861, but not in either strain 4496 or the lineage A strains. Therefore, the region is unlikely to be responsible for the differences in virulence between serotype 1 isolates in this study. In addition, ARs 11, 30 and 34, which encode the pilus, pneumococcal collagen-like protein (PclA) and PsrP, respectively, have been implicated in virulence [10], [16], [19], [69]. However, AR 11 was absent in all six isolates, and AR 34 was present in the intermediately virulent isolates and strain 1861, but not in the non-invasive isolates or strain 4496. AR 30 was present only in the intermediately virulent isolates. Therefore, these regions are unlikely to be important for the differences in virulence between the isolates in this study. In addition to the pilus encoded within the *rlrA* islet, there is a second pilus (PI-2), which has been reported to be present in emerging serotypes such as 1, 2, 7F and 19A and is homologous to *SPT_1056–1064* in Taiwan^19F^-14 [70]. However, genome sequencing and PCR (data not shown) showed that PI-2 is present in the non-invasive and intermediately virulent isolates, but absent in the highly virulent isolates. Therefore, possession of PI-2 is not required for the heightened virulence of strains 1861 and 4496.

**Table 4:** Other notable regions not consistently associated with a virulence profile in this study.

AR*	Putative annotation/function of key gene(s) in region	Homologous ORFs		Serotype 1 isolates					
		TIGR4	D39	1	2	3415	5482	1861	4496
5	Hypothetical proteins	0296–0298	-	−	−	−	−	−	−
6	β glucosidase, PTS – metabolic	0300–0310	0276–0283	+	+	+	+	−	−
9	Mevalonate pathway - metabolism	0382–0387	0347–0390	+	+	+	+	+	+
10	Mannitol PTS	0394–0399	0360–0364	+	+	+	+	−	−
11	*RlrA* islet - adherence	0461–0468	-	−	−	−	−	−	−
15	β-galactosidase – metabolism	0643–0648	0559–0562	+	+	+	+	+	+
16	Zinc metalloproteinase B	0664–0666	-	+	+	+	+	+	+
19	Type I restriction modification system	0887–0890	-	+	+	+	+	+	+
20	Amino acid metabolism	0918–0923	0811–0816	+	+	+	+	+	+
21	CelA - competence	0949–0954	0839–0843	+	+	+	+	+	+
27	V-type sodium ATP synthase	1315–1331	-	−	−	+	+	−	−
29	ABC transporter	1432–1442	1261–1272	+	+	+	+	+	+
30	Collagen-like protein, PclA – adherence	-	1376–1377	+	+	+	+	−	−
31	Ribulose PTS - uptake of ribulose	1612–1620	-	−	−	−	−	+	−
34	PsrP-secAY2A2 - adherence	1755–1772	-	−	−	+	+	−	+
39	Type II restriction modification system	1930–1936	1731–1736	+	+	+	+	+	+
-	PI-2 adherence	-	-	+	+	+	+	−	−

*AR designated by Bloomberg *et al.* [14].

+, Region present; −, Region absent; Determined from genome sequence of strains 1 and 1861, and by CGH for strains 2, 3415, 5482 and 4496. Presence of PI-2 was determined by PCR.

### Differential *in vivo* expression of key virulence-associated genes

Genes associated with heightened virulence (Tables 2 and 3) were selected for *in vivo* gene expression comparisons to identify genes that exhibit niche-specific changes in expression. Expression analysis was performed on nasal lavage, blood and lung samples of strain 1861- and 4496-infected mice (Figure 2), using qRT-PCR. Expression of the endolysin, *nplT*, 3HIBDH and iron/lead permease was elevated in the blood and lungs of both strain 1861- and 4496-infected mice, when compared to the nasopharyngeal lavage fluid (Table 5), which suggests that the products of these genes are more important in the blood and lungs than the nasopharyngeal surface. Interestingly, since endolysin activity has been shown to be required for PblB surface expression in *S. mitis* [33], surface expression of this potential adherence factor could be indirectly elevated in the lungs and blood due to endolysin activity, despite little difference in *pblB* expression between niches. In PPI-1v, 3HIBDH is part of an operon that also encodes PDT and *galE* (data not shown), which implies that the expression of all three of these enzymes is elevated in the lungs and blood compared to the nasopharyngeal surface. While the activities of these genes remain to be confirmed experimentally, the products of these genes could provide a survival advantage and facilitate disease. Interestingly, expression of the iron/lead permease was greatest in the blood followed by the lungs and lowest on the nasopharyngeal surface, which suggests that this transporter is more important in the blood and lungs than on the nasopharyngeal mucosa. Expression of the major facilitator transporter in PPI-1v and the glycoside hydrolase in region 8 was significantly greater in the blood than either the lungs or nasopharyngeal surface. However, since the substrate of the major facilitator is unknown it is not clear whether the expression of this gene provides a survival advantage in this niche. As the glycoside hydrolase is encoded immediately downstream of the putative ABC transporter in region 8, and no expression-attenuating secondary structures were predicted in the intervening sequence, it is likely that the expression of this enzyme reflects the expression of the ABC transporter. Expression of the sodium-dependent permease, *pezAT* and biotin carboxylase expression in PPI-1v did not appear to be niche-specific since changes in the expression of these genes were not consistent between the two strains.

**Table 5:** Relative expression of selected virulence associated genes between different niches of the mouse.

Gene	Region+	Expression∧					
		Blood vs. nose		Lungs vs. nose		Blood vs. lungs	
		1861	4496	1861	4496	1861	4496
*pblB*	1	+1.22*^ns^*	+1.79*^ns^*	+1.17*^ns^*	−2.43^b^	+1.04*^ns^*	+1.36*^ns^*
Endolysin	1	+1311.20* c	+221.32* c	+1028.74* c	+220.30* c	+1.27*^ns^*	1.00*^ns^*
Na+ dep. transporter	2	−1.28*^ns^*	−1.28*^ns^*	−3.15^c^	+1.54	+2.47^b^	−1.97*^ns^*
*nplT*	3	+55.72^c^	+42.62^c^	+28.38* c	+69.07* c	+1.96*^ns^*	−1.62*^ns^*
*pezAT*	3	+1.06*^ns^*	+11.71^c^	−1.70*^ns^*	+1.14*^ns^*	+1.80*^ns^*	+10.29^c^
3HIBDH	4	+64.59* c	+432.53* c	+115.89* c	+97.46* c	−1.79*^ns^*	+4.44^c^
Biotin carboxylase	4	−1.63*^ns^*	+97.01^c^	−2.61^c^	+9.78^c^	+1.60*^ns^*	+9.92^c^
Major facilitator	4	+3.27^c^	+10.95^c^	+1.08*^ns^*	+2.04^c^	+3.03^c^	+5.36^c^
Fe^2+^/Pb^2+^permease	6	+5.92^c^	+24.36^c^	+2.17^c^	+12.10^c^	+2.73^a^	+2.01^a^
Gly. Hydrolase	8	+29.18* c	369.65* c	-#	-#	+624.55* c	+25.22^c^

*Indicates where the target mRNA was below the limit of detection in nasal wash-derived RNA.

# Indicates where the target mRNA was below the limit of detection in both samples.

∧ The values represent the relative amounts of mRNA in the first niche compared to the second.

Results of statistical analysis using *t*-test: ns, not significant (includes values <2);

a , *P*<0.05;

b , *P*<0.01;

c , *P*<0.001.

+ As numbered in Table 2.

### *In vivo* competition between D39 PPI-1 mutants replacing the endogenous version of the region with the regions of strain 1 and strain 1861

**Figure 5:**
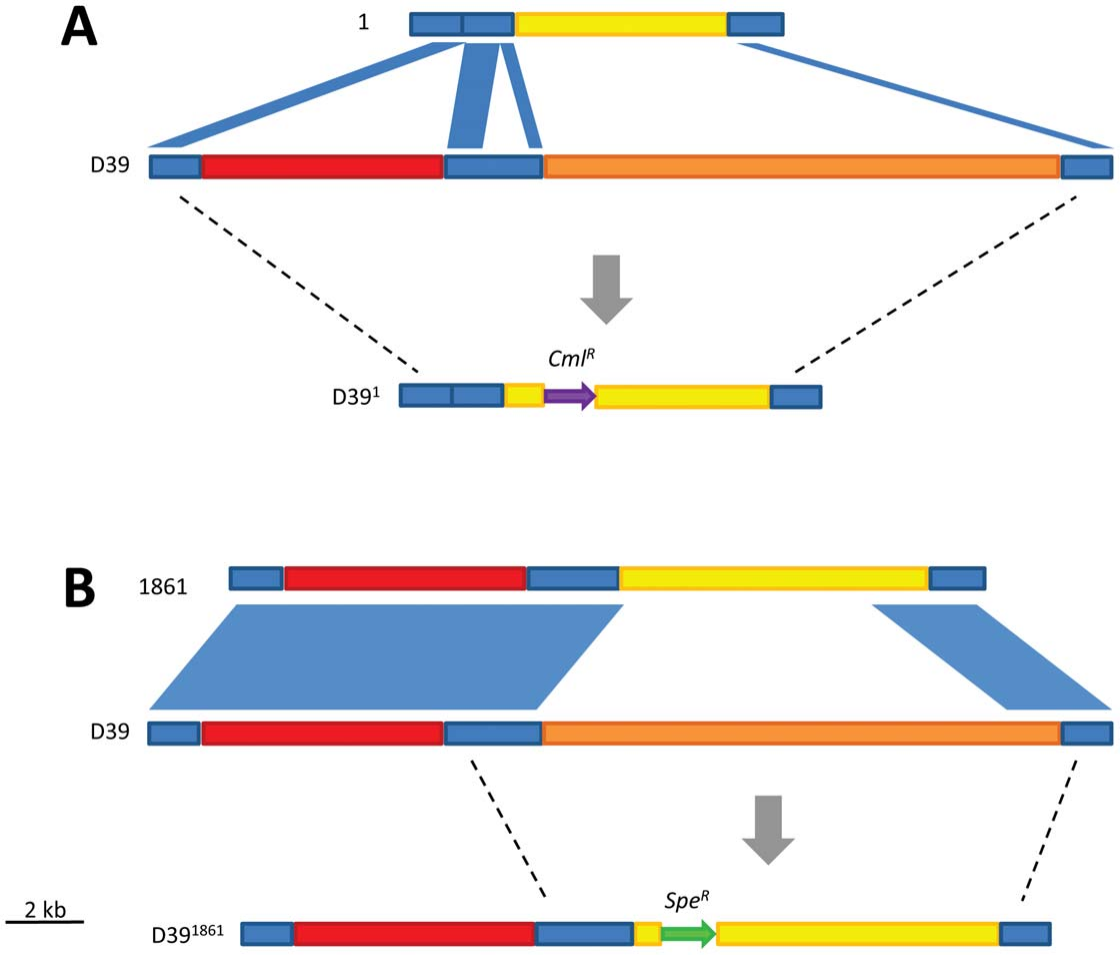
Construction of PPI-1v derivatives in D39 representing strains 1 and 1861. The PPI-1v derivatives D39^1^ (A) and D39^1861^ (B) were constructed to reflect the organization of the region in the lineage A strains and highly virulent isolates, respectively. PPI-1v alignments between the D39 wild-type region and the region in strains 1 (A) and 1861 (B) indicate nucleotide sequence identity exceeding 90%, and highlights the non-homologous wild-type sequence that was replaced. The approximate sites of homologous recombination were subsequently determined by sequencing and are indicated by broken lines. The PPI-1v accessory region of strains 1 and 1861 are colored yellow and the wild-type PPI-1v accessory region is colored orange. The relative position of the antibiotic resistance genes conferring resistance against chloramphenicol (*cml^R^*) and spectinomycin (*spe^R^*) are indicated in the final mutant.

**Figure 6:**
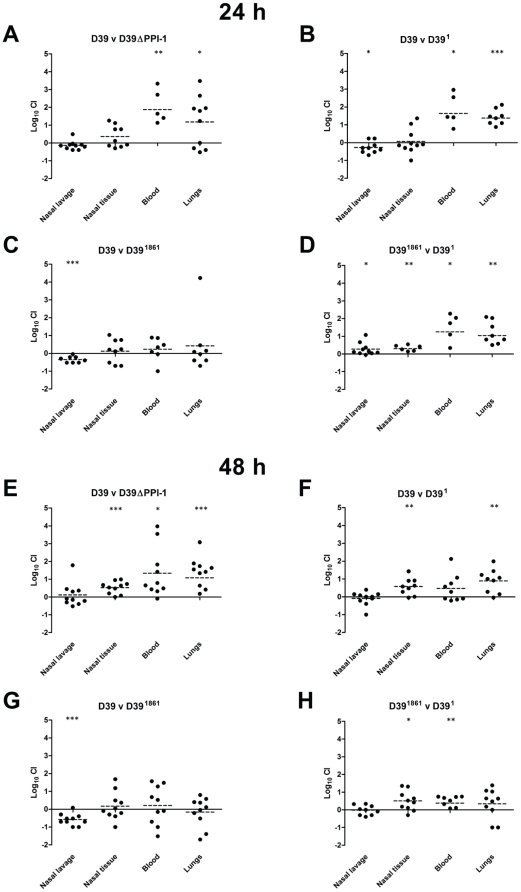
Competition between PPI-1v derivatives and wild type in the nasopharynx, blood and lungs. Competitive index (CI) within nasal lavage, nasal tissue, blood and lung samples of individual mice are indicated at 24 h (A–D) and 48 h (E–H) post-challenge. CIs are expressed as log-transformed ratios of the first strain relative to the second strain for mice where pneumococci CFU were recovered from the relevant niche. Values are pooled from two replicate experiments that used 5 mice per group at each time point. Statistical differences between the log-transformed geometric mean CI and a hypothetical value of 0 (ratio of 1∶1) in each niche were analyzed using the one-sample *t*-test (*, *P*<0.05; **, *P*<0.01; ***, *P*<0.001).

Since the content of PPI-1v varies between the highly virulent isolates and the four less virulent isolates, and the expression of a number of PPI-1v genes was favored in niches associated with disease, it was decided to confirm the role of this region in virulence determination by mutagenesis. However, a significant roadblock to such experiments was the inability to genetically transform the serotype 1 isolates in this study, despite numerous attempts using various known transformation protocols. Therefore, it was decided to construct PPI-1v derivatives in the easily transformable laboratory strain D39 [71], which has a different version of PPI-1v compared to strain 1 and 1861. Derivatives of PPI-1v were constructed and used to replace the endogenous region with the version of the region in the highly virulent isolates (designated D39^1861^) and the version in the non-invasive isolates (designated D39^1^). PPI-1v in D39^1^ was also representative of that present in the intermediately virulent isolates. In addition, a PPI-1v deletion mutant (D39ΔPPI-1) was constructed to examine the contribution of the wild-type D39 version of PPI-1v to virulence. In order to obtain a D39ΔPPI-1 knockout, an intermediate mutant lacking *pezT* (D39ΔPezT) was constructed by replacing *SPD_0931–0951* with *erm^R^*, as simultaneous deletion of *pezA* and *pezT* has been reported to be lethal [44]. The subsequent D39ΔPPI-1 mutant was constructed from D39ΔPezT by replacing *SPD_0927*–*0930* with *cml^R^*. D39^1^ was also constructed from D39ΔPezT by replacing the remaining non-homologous D39 PPI-1v sequence (*SPD_0927*–*0930*) with strain 1 sequence (*SPINV104_09310–09190*) (Fig. 5A). D39^1861^ was constructed by replacing the non-homologous D39 PPI-1v sequence (*SPD_0936–0951*) with strain 1861 PPI-1v sequence (*SPP_1056–1072*) (Fig. 5B). Expression of 1861-derived PPI-1v genes in D39^1861^ was confirmed *in vitro* by qRT-PCR (data not shown). In addition, there was no detectable difference in *in vitro* growth rate between the mutants and D39 (data not shown). The ability of the mutants and wild-type to cause disease was subsequently compared in mice using mixed infections using the combinations D39 vs. D39ΔPPI-1, D39 vs. D39^1^, D39 vs. D39^1861^ and D39^1861^ vs. D39^1^. Data were obtained from the nasal lavage fluid, nasal tissue, lungs and blood at both 24 h and 48 h post-challenge. At 24 h both D39ΔPPI-1 and D39^1^ were less competitive than the wild type in both the lungs and blood (Fig. 6A and 6B). However, D39^1^ was more competitive than the wild-type at the nasopharyngeal surface. In contrast, there was no significant difference between D39^1861^ and the wild type in the blood, lungs or nasal tissue (Fig. 6C). However, D39^1861^ was more competitive than the wild type on the nasopharyngeal mucosa. Interestingly, D39^1861^ was more competitive than D39^1^ in all four niches (Fig. 6D). Furthermore, at 48 h the wild type was more competitive than D39ΔPPI-1 in the nasal tissue, lungs and blood (Fig. 6E). In contrast, D39^1^ was less competitive than the wild type in the nasal tissue and the lungs, but was equally competitive in the blood at 48 h (Fig. 6F). Similar to 24 h, D39^1816^ was as competitive as the wild-type in the nasal tissue, blood and lungs, but more competitive on the nasopharyngeal surface at 48 h (Fig. 6G). D39^1861^ was also more competitive than D39^1^ in the nasal tissue and blood, but equally competitive in the lungs and at the nasopharyngeal surface (Fig. 6H). In summary, it is clear that PPI-1v plays a role in virulence in a D39 background, and that it is possible that PezAT is important as has been previously reported [42]. However, the importance of other components of PPI-1v in virulence cannot be ruled out. In addition, while the endogenous version of PPI-1v is required for wild-type virulence in D39, it is also true that in a D39 background, PPI-1v from the highly virulent serotype 1 strains was also more competitive than PPI-1v from the non-invasive and intermediately virulent serotype 1 isolates in a number of niches, which correlates with differences in virulence between the isolates in both mice and humans. Therefore, while it is possible that the relative impact of PPI-1v on virulence could be different in a D39 background than in the serotype 1 isolates themselves, the correlation between the virulence of the wild-type strains, the niche-specific changes in expression of PPI-1v genes and *in vivo* competition between PPI-1v D39 mutants is compelling.

This study aimed to identify serotype-independent virulence determinants within the genomes of a selection of serotype 1 isolates with wide-ranging virulence in both mice and humans. In particular, isolates closely related to the hypervirulent clones responsible for epidemic IPD were found to readily invade and survive in the blood, whereas non-invasive clones isolated from episodes of asymptomatic serotype 1 carriage in remote Indigenous Australian communities were only able to colonize the nasopharynx in mice. A number of regions >1 kb in size were present in the genomes of only the highly virulent isolates, which included a phage genome with putative adherence factors, a number of putative metabolic enzymes, an ABC transporter, an ion transporter as well as a number of potential stress-responsive transcriptional regulators. In particular, the expression of some metabolic enzymes, transporters and adherence factors appeared to exhibit preferential expression in niches associated with IPD. In addition, the various versions of PPI-1v were shown to impact on virulence differently and suggests that this region is at least partly responsible for the greater virulence of highly virulent isolates compared to less virulent isolates. PPI-1v appears to be a highly variable AR within the pneumococcal genome that alters the survival of the bacterium within the host in a content-dependent fashion.

## Materials and Methods

### Ethics statements

This study was conducted in compliance with the Australian code of practice for the care and use of animals for scientific purposes (7th Edition 2004) and the South Australian Animal Welfare Act 1985. All animal experiments were approved by the Animal Ethics Committee of the University of Adelaide (Project Number: S- 86-2006). Written consent was obtained for studies of human specimens and ethics approval was obtained for further molecular analyses from the Human Research Ethics Committe of the Menzies School of Health Research and Department of Health and Families.

### Strains and media

The *S. pneumoniae* serotype 1 clinical strains used in this study that were of Indigenous Australian origin included non-invasive isolates (strains Menzies^1^-1 [ST304] and Menzies^1^-2 [ST304]), and invasive isolates (strains Menzies^1^-3415 [ST227], Menzies^1^-5482 [ST227] and Menzies^1^-1861) and were provided by the Royal Darwin Hospital Pathology Services. An invasive serotype 1 isolate (strain WCH4496) was of non-Indigenous origin and was obtained from the Women's and Children's Hospital, North Adelaide, Australia. The virulent serotype 2 strain, D39 (NCTC 7466) was also used in this study. The sequence type (ST) of serotype 1 strains was determined by MLST as described in Enright & Spratt (1998) and in accordance with the instructions at http://spneumoniae.mlst.net. Opaque-phase variants of all strains selected on Todd-Hewitt broth supplemented with 1% yeast extract (THY)-catalase plates [72] were used in all animal experiments. Before infection, the bacteria were grown in serum broth (SB) (nutrient broth [10 g/l peptone (Oxoid), 10 g/l Lab Lemco powder (Oxoid) and 5 g/l NaCl] and 10% [v/v] donor horse serum) to an optical density at 600 nm (OD_600_) of 0.16, which approximates 1×10^8^ CFU/ml.

### PCR

Chromosomal DNA for PCR was extracted and purified using the Wizard genomic DNA purification kit (Promega Corporation, Madison, WI), with the exception of cell lysis, which was performed by incubating cells at 37°C for 10 min with 0.1% (w/v) sodium deoxycholate. PCR reactions were performed using a G-STORM GS482 thermal cycler (Gene Technologies, UK). Standard reactions were performed using *Taq* DNA polymerase (Roche Diagnostics, Basel Switzerland) according to the manufacturer's instructions. The Expand™ Long template or High fidelity PCR systems were used when high fidelity amplification was required. Overlap-extension PCR was carried out essentially as previously described [73], [74], using the Expand™ Long Template PCR system. DNA sequencing reactions were carried out using the BigDye® Terminator v3.1 Cycle Sequencing kit (Applied Biosystems, CA, USA).

### Animal studies

Inbred 5- to 6-week old female Balb/c mice were used in i.p. challenge experiments and outbred 5- to 6-week old female CD1 (Swiss) mice were used in i.n. challenge experiments. For i.p. challenge experiments groups of 5 mice were used. Mice were challenged i.p with 100 µl of bacterial suspension containing approximately 1×10^4^ CFU in SB. The challenge dose was confirmed retrospectively by serial dilution and plating on blood agar. Mice were monitored for signs of illness over 9 days and were euthanized when moribund. Blood was taken from euthanized mice and plated on blood agar to confirm the presence of *S. pneumoniae* in the blood. For i.n. challenge, groups of 10 mice were anaesthetized by i.p. injection of pentobarbital sodium (Nembutal; Rhone-Merieux) at a dose of 66 µg per g of body weight and challenged with 50 µl of bacterial suspension containing approximately 1×10^7^ CFU in SB. The challenge dose was confirmed retrospectively as described above for i.p. challenge. For quantification of pneumococci and gene expression analysis in infected mouse tissues, groups of 30 mice for strains Menzies^1^-1861 and WCH4496 and a group of 20 mice for strain Menzies^1^-1 were challenged via the i.n. route with anesthesia as described above. At 48 h and 96 h, the mice were euthanized by CO_2_ asphyxiation, and nasal lavage, nasal tissue, lung and blood samples were processed as previously described [75], [76]. A 40-µl aliquot of each sample was serially diluted in phosphate-buffered saline and plated on blood agar to enumerate pneumococci present in each niche and to determine the presence, if any, of contaminating microflora. Blood plates were incubated at 37°C in 95% air, 5% CO_2_ overnight. Samples were then stored at −80°C until further processing was performed. In addition, a 400-µl aliquot of blood and homogenized nasal and lung tissue was also harvested from each mouse for extraction of prokaryotic RNA. For *in vivo* competition experiments, two replicate experiments with groups of ten mice per competition group were challenged i.n. (as described above) with a mixed culture of approximately 5×10^6^ CFU per strain. The competitive index (CI) within nasal wash, nasal tissue, blood and lung samples was determined at 24 h and 48 h post-challenge on selective media by calculating the ratio of wild-type to mutant or mutant to mutant as required, relative to the input ratio. As the CI values were log transformed, a value close to 0 is expected if strains compete equally.

### Extraction of total RNA from infected host tissues

RNA was extracted from host tissues, purified and enriched for bacterial RNA essentially as described previously [75], [77]. In this experiment bacterial RNA was pooled from the same 4 mice per niche.

### Linear amplification of total RNA

Bacterial-RNA samples were amplified using a RNA linear amplification kit SenseAMP (Genisphere), as described previously [75], [77].

### Real-time relative qRT-PCR

The abundance of mRNAs of the genes listed in Table 5 present in amplified RNA recovered from pneumococci harvested from all niches was measured by real-time quantitative RT-PCR (qRT-PCR). Gene specific primers were designed using OligoPerfect™ software (Invitrogen), and primers specific for 16S rRNA were used as internal controls for data normalization (Table S1). qRT-PCR was performed using a LightCycler® 480 II (Roche) using the Superscript III One-step RT-PCR kit (Invitrogen) according to the manufacturer's instructions. Quantitative differences for each transcript were calculated using the 2^−ΔΔCT^ method [78]. Expression data are expressed as a relative increase/decrease between niches.

### Comparative Genomic Hybridization

Comparative genomic hybridization (CGH) experiments were performed on whole genome *S. pneumonia* PCR microarrays based on TIGR4 and R6 annotations. Microarray slides were obtained from the Bacterial Microarray Group at St George's Hospital, University of London. The microarray design is available in BμG@Sbase (Accession No. A-BUGS-14; http://bugs.sgul.ac.uk/A-BUGS-14) and ArrayExpress (Accession No. A-BUGS-14). *S. pneumoniae* DNA for CGH was extracted and purified using a phenol extraction method as described previously [79]. DNA (10.5 µg in 100 µl) was digested with *Sau*3AI (New England Biolabs [NEB], MA, USA), and purified using the Qiagen MinElute® PCR Purification kit. Thereafter, 20 µl of purified digest was labeled using the Genisphere Array 900 DNA™ DNA labeling kit for Microarrays (Genisphere, PA, USA) for each of the dyes used (Alexa Fluor 555 and Alex Fluor 647). Slides were incubated overnight in a dark humidified chamber at 65°C, washed in a 3-step process (15 min with 2× SSC, 0.03% (v/v) SDS, at 65°C; 15 min with 1× SSC, RT; 15 min with 0.2× SSC, at RT) and dried. Slides were scanned using GenePix® Pro 6.0 software (Axon). CGH was performed in pairs of strains with the same virulence phenotype per slide (1 & 2, 3415 & 5482, 1861 & 4496). Approximately equal fluorescence per spot on the array between channels on one slide indicated presence in both strains of the same phenotype, unequal fluorescence indicated presence only in one strain of the same virulence phenotype, and fluorescence not significantly different from the background indicated absence in both strains of the same virulence phenotype.

### Genomic sequencing

Sequencing and genome assembly were performed by Geneworks (Thebarton, Adelaide, Australia) using chromosomal DNA prepared as described for CGH, using an Illumina Genome Analyzer *II* (California, USA) and Lasergene® 8 software (DNASTAR Inc, WI, USA). The sequenced 35-bp reads were assembled against the P1301 (serotype 1, ST303) genome (Genbank accession no. CP000920). The assembly of the strain 1 reads generated a 1,947,650-bp consensus sequence (92.22% of P1031) with an average read depth of 35.72 reads. The assembly of the strain 1861 reads generated a 2,105,218-bp consensus sequence (99.68% of P1031) with an average read depth of 43.66 reads. Unassembled sequences were subsequently assembled *de novo* into 103 and 62 contigs >300-bp in size from strain 1 and 1861, respectively. While many smaller contigs of boneyard sequence were assembled, these largely represented gaps in sequence assembly due to sequence variation below the 80% sequence identity cutoff. The genome sequence of INV104 (Genbank accession no. FQ312030), ATCC 700669 (Genbank accession no. FM211187), G54 (Genbank accession no. CP001015) and D39 (Accession no. NC_008533) were used in comparisons. Alignments were performed using the Artemis Comparison Tool (ACT) [80]. The sequences of strains 1 and 1861 were submitted to the sequence read archive at NCBI and have accession numbers SPX030816.2 and SPX030825.2, respectively.

### Construction of PPI-1 variable region mutants

Mutants were constructed using the primers in Table S1. Mutants requiring the deletion of *pezAT* were performed in two steps. D39ΔPPI-1 and D39^1^ were constructed from D39ΔPezT. The construct for D39ΔPezT was generated by overlap extension PCR, performed essentially as described below, from products amplified using primers *a – aq* and *ec – g* for the flanking products and J214–J215 for the amplification of *erm^R^* from pVA891 [81]. The construct for D39ΔPPI-1 was generated by restriction endonuclease treatment and subsequent ligation using primers *t – ed* and *ee – g* for the flanking products amplified from D39 and RHcatF – RHcatR for amplification of *cml^R^*. The construct for D39^1^ was generated by restriction endonuclease treatment and subsequent ligation using primers *af – ei* and *ej – g* for the flanking products amplified from strain 1 DNA and *cml^R^*. The construct for D39^1861^ was generated by restriction endonuclease treatment and subsequent ligation using primers *ef – eg* and *eh – c* for the flanking products amplified from strain 1861 DNA and J293a–J254a for amplification of *spe^R^*. Generation of competent *S. pneumoniae* cells and subsequent transformation was performed using the complete transformation medium (CTM) method [82], [83].

### Statistical analyses

Differences in median survival times and differences in the geometric mean number of pneumococci in each niche between groups were analyzed by the unpaired *t*-test (two tailed). Differences in the relative expression levels of genes between niches were performed using the unpaired *t*-test (two tailed). Differences between the competitive index of a test sample and CI = 1 were analyzed using log-transformed values by one-sample *t*-test. All analyses were performed using GraphPad Prism version 5.01. *P*<0.05 was considered significant.

## Supporting Information

Table S1 **List of oligonucleotides used in this study.**
(DOC)

Table S2 **List of genes present in ARs associated with hypervirulence.**
(XLS)

## References

[1] O'Brien KL, Wolfson LJ, Watt JP, Henkle E, Deloria-Knoll M (2009). Burden of disease caused by *Streptococcus pneumoniae* in children younger than 5 years: global estimates.. Lancet.

[2] Brueggemann AB, Griffiths DT, Meats E, Peto T, Crook DW (2003). Clonal relationships between invasive and carriage *Streptococcus pneumoniae* and serotype- and clone-specific differences in invasive disease potential.. J Infect Dis.

[3] Brueggemann AB, Peto TE, Crook DW, Butler JC, Kristinsson KG (2004). Temporal and geographic stability of the serogroup-specific invasive disease potential of *Streptococcus pneumoniae* in children.. J Infect Dis.

[4] Sandgren A, Albiger B, Orihuela CJ, Tuomanen E, Normark S (2005). Virulence in mice of pneumococcal clonal types with known invasive disease potential in humans.. J Infect Dis.

[5] Sandgren A, Sjostrom K, Olsson-Liljequist B, Christensson B, Samuelsson A (2004). Effect of clonal and serotype-specific properties on the invasive capacity of *Streptococcus pneumoniae*.. J Infect Dis.

[6] Sjostrom K, Spindler C, Ortqvist A, Kalin M, Sandgren A (2006). Clonal and capsular types decide whether pneumococci will act as a primary or opportunistic pathogen.. Clin Infect Dis.

[7] Nunes S, Sa-Leao R, Pereira LC, Lencastre H (2008). Emergence of a serotype 1 *Streptococcus pneumoniae* lineage colonising healthy children in Portugal in the seven-valent conjugate vaccination era.. Clin Microbiol Infect.

[8] Smith-Vaughan H, Marsh R, Mackenzie G, Fisher J, Morris PS (2009). Age-specific cluster of cases of serotype 1 *Streptococcus pneumoniae* carriage in remote indigenous communities in Australia.. Clin Vaccine Immunol.

[9] Silva NA, McCluskey J, Jefferies JM, Hinds J, Smith A (2006). Genomic diversity between strains of the same serotype and multilocus sequence type among pneumococcal clinical isolates.. Infect Immun.

[10] Obert C, Sublett J, Kaushal D, Hinojosa E, Barton T (2006). Identification of a Candidate *Streptococcus pneumoniae* core genome and regions of diversity correlated with invasive pneumococcal disease.. Infect Immun.

[11] Tettelin H, Nelson KE, Paulsen IT, Eisen JA, Read TD (2001). Complete genome sequence of a virulent isolate of *Streptococcus pneumoniae*.. Science.

[12] Hiller NL, Janto B, Hogg JS, Boissy R, Yu S (2007). Comparative genomic analyses of seventeen *Streptococcus pneumoniae* strains: insights into the pneumococcal supragenome.. J Bacteriol.

[13] Bruckner R, Nuhn M, Reichmann P, Weber B, Hakenbeck R (2004). Mosaic genes and mosaic chromosomes-genomic variation in *Streptococcus pneumoniae*.. Int J Med Microbiol.

[14] Blomberg C, Dagerhamn J, Dahlberg S, Browall S, Fernebro J (2009). Pattern of accessory regions and invasive disease potential in *Streptococcus pneumoniae*.. J Infect Dis.

[15] Aguiar SI, Serrano I, Pinto FR, Melo-Cristino J, Ramirez M (2008). The presence of the pilus locus is a clonal property among pneumococcal invasive isolates.. BMC Microbiol.

[16] Barocchi MA, Ries J, Zogaj X, Hemsley C, Albiger B (2006). A pneumococcal pilus influences virulence and host inflammatory responses.. Proc Natl Acad Sci U S A.

[17] Basset A, Trzcinski K, Hermos C, O'Brien KL, Reid R (2007). Association of the pneumococcal pilus with certain capsular serotypes but not with increased virulence.. J Clin Microbiol.

[18] Orihuela CJ (2009). Role played by psrP-secY2A2 (accessory region 34) in the invasive disease potential of *Streptococcus pneumoniae*.. J Infect Dis.

[19] Shivshankar P, Sanchez C, Rose LF, Orihuela CJ (2009). The *Streptococcus pneumoniae* adhesin PsrP binds to Keratin 10 on lung cells.. Mol Microbiol.

[20] Sjostrom K, Blomberg C, Fernebro J, Dagerhamn J, Morfeldt E (2007). Clonal success of piliated penicillin nonsusceptible pneumococci.. Proc Natl Acad Sci U S A.

[21] Sanchez CJ, Shivshankar P, Stol K, Trakhtenbroit S, Sullam PM (2010). The pneumococcal serine-rich repeat protein is an intra-species bacterial adhesin that promotes bacterial aggregation *in vivo* and in biofilms.. PLoS Pathog.

[22] Hyams C, Yuste J, Bax K, Camberlein E, Weiser JN (2010). *Streptococcus pneumoniae* resistance to complement-mediated immunity is dependent on the capsular serotype.. Infect Immun.

[23] Weinberger DM, Trzcinski K, Lu YJ, Bogaert D, Brandes A (2009). Pneumococcal capsular polysaccharide structure predicts serotype prevalence.. PLoS Pathog.

[24] Melin M, Trzcinski K, Antonio M, Meri S, Adegbola R (2010). Serotype related variation in susceptibility to complement deposition and opsonophagocytosis among clinical isolates of *Streptococcus pneumoniae*.. Infect Immun.

[25] Melin M, Trzcinski K, Meri S, Kayhty H, Vakevainen M (2010). The capsular serotype of *Streptococcus pneumoniae* is more important than the genetic background for resistance to complement.. Infect Immun.

[26] Brueggemann AB, Spratt BG (2003). Geographic distribution and clonal diversity of *Streptococcus pneumoniae* serotype 1 isolates.. J Clin Microbiol.

[27] Antonio M, Hakeem I, Awine T, Secka O, Sankareh K (2008). Seasonality and outbreak of a predominant *Streptococcus pneumoniae* serotype 1 clone from The Gambia: expansion of ST217 hypervirulent clonal complex in West Africa.. BMC Microbiol.

[28] Leimkugel J, Adams Forgor A, Gagneux S, Pfluger V, Flierl C (2005). An outbreak of serotype 1 *Streptococcus pneumoniae* meningitis in northern Ghana with features that are characteristic of *Neisseria meningitidis* meningitis epidemics.. J Infect Dis.

[29] Yaro S, Lourd M, Traore Y, Njanpop-Lafourcade BM, Sawadogo A (2006). Epidemiological and molecular characteristics of a highly lethal pneumococcal meningitis epidemic in Burkina Faso.. Clin Infect Dis.

[30] van der Ploeg JR (2010). Genome sequence of the temperate bacteriophage PH10 from *Streptococcus oralis*.. Virus Genes.

[31] Romero P, Croucher NJ, Hiller NL, Hu FZ, Ehrlich GD (2009). Comparative genomic analysis of ten *Streptococcus pneumoniae* temperate bacteriophages.. J Bacteriol.

[32] Romero P, Garcia E, Mitchell TJ (2009). Development of a prophage typing system and analysis of prophage carriage in *Streptococcus pneumoniae*.. Appl Environ Microbiol.

[33] Mitchell J, Siboo IR, Takamatsu D, Chambers HF, Sullam PM (2007). Mechanism of cell surface expression of the *Streptococcus mitis* platelet binding proteins PblA and PblB.. Mol Microbiol.

[34] Bensing BA, Rubens CE, Sullam PM (2001). Genetic loci of *Streptococcus mitis* that mediate binding to human platelets.. Infect Immun.

[35] Mitchell J, Sullam PM (2009). *Streptococcus mitis* phage-encoded adhesins mediate attachment to {alpha}2–8-linked sialic acid residues on platelet membrane gangliosides.. Infect Immun.

[36] Seo HS, Xiong YQ, Mitchell J, Seepersaud R, Bayer AS (2010). Bacteriophage lysin mediates the binding of *Streptococcus mitis* to human platelets through interaction with fibrinogen.. PLoS Pathog.

[37] Berry AM, Lock RA, Hansman D, Paton JC (1989). Contribution of autolysin to virulence of *Streptococcus pneumoniae*.. Infect Immun.

[38] Berry AM, Paton JC (2000). Additive attenuation of virulence of *Streptococcus pneumoniae* by mutation of the genes encoding pneumolysin and other putative pneumococcal virulence proteins.. Infect Immun.

[39] Canvin JR, Marvin AP, Sivakumaran M, Paton JC, Boulnois GJ (1995). The role of pneumolysin and autolysin in the pathology of pneumonia and septicemia in mice infected with a type 2 pneumococcus.. J Infect Dis.

[40] Martner A, Skovbjerg S, Paton JC, Wold AE (2009). *Streptococcus pneumoniae* autolysis prevents phagocytosis and production of phagocyte-activating cytokines.. Infect Immun.

[41] Stroeher UH, Kidd SP, Stafford SL, Jennings MP, Paton JC (2007). A pneumococcal MerR-like regulator and S-nitrosoglutathione reductase are required for systemic virulence.. J Infect Dis.

[42] Brown JS, Gilliland SM, Spratt BG, Holden DW (2004). A locus contained within a variable region of pneumococcal pathogenicity island 1 contributes to virulence in mice.. Infect Immun.

[43] Croucher NJ, Walker D, Romero P, Lennard N, Paterson GK (2009). Role of conjugative elements in the evolution of the multidrug-resistant pandemic clone *Streptococcus pneumoniae* Spain23F ST81.. J Bacteriol.

[44] Khoo SK, Loll B, Chan WT, Shoeman RL, Ngoo L (2007). Molecular and structural characterization of the PezAT chromosomal toxin-antitoxin system of the human pathogen *Streptococcus pneumoniae*.. J Biol Chem.

[45] Hollands A, Aziz RK, Kansal R, Kotb M, Nizet V (2008). A naturally occurring mutation in *ropB* suppresses SpeB expression and reduces M1T1 group A streptococcal systemic virulence.. PLoS One.

[46] Poulsen K, Reinholdt J, Kilian M (1996). Characterization of the *Streptococcus pneumoniae* immunoglobulin A1 protease gene (*iga*) and its translation product.. Infect Immun.

[47] Oggioni MR, Memmi G, Maggi T, Chiavolini D, Iannelli F (2003). Pneumococcal zinc metalloproteinase ZmpC cleaves human matrix metalloproteinase 9 and is a virulence factor in experimental pneumonia.. Mol Microbiol.

[48] Chiavolini D, Memmi G, Maggi T, Iannelli F, Pozzi G (2003). The three extra-cellular zinc metalloproteinases of *Streptococcus pneumoniae* have a different impact on virulence in mice.. BMC Microbiol.

[49] Kilian M, Mestecky J, Schrohenloher RE (1979). Pathogenic species of the genus *Haemophilus* and *Streptococcus pneumoniae* produce immunoglobulin A1 protease.. Infect Immun.

[50] Weiser JN, Bae D, Fasching C, Scamurra RW, Ratner AJ (2003). Antibody-enhanced pneumococcal adherence requires IgA1 protease.. Proc Natl Acad Sci U S A.

[51] Kilian M, Reinholdt J, Lomholt H, Poulsen K, Frandsen EV (1996). Biological significance of IgA1 proteases in bacterial colonization and pathogenesis: critical evaluation of experimental evidence.. APMIS.

[52] Wani JH, Gilbert JV, Plaut AG, Weiser JN (1996). Identification, cloning, and sequencing of the immunoglobulin A1 protease gene of *Streptococcus pneumoniae*.. Infect Immun.

[53] Camilli R, Pettini E, Del Grosso M, Pozzi G, Pantosti A (2006). Zinc metalloproteinase genes in clinical isolates of *Streptococcus pneumoniae*: association of the full array with a clonal cluster comprising serotypes 8 and 11A.. Microbiology.

[54] Henderson-Begg SK, Roberts AP, Hall LM (2009). Diversity of putative Tn5253-like elements in *Streptococcus pneumoniae*.. Int J Antimicrob Agents.

[55] Ayoubi P, Kilic AO, Vijayakumar MN (1991). Tn*5253*, the pneumococcal omega (*cat tet*) BM6001 element, is a composite structure of two conjugative transposons, Tn*5251* and Tn*5252*.. J Bacteriol.

[56] Munoz-Najar U, Vijayakumar MN (1999). An operon that confers UV resistance by evoking the SOS mutagenic response in streptococcal conjugative transposon Tn*5252*.. J Bacteriol.

[57] Smith BT, Walker GC (1998). Mutagenesis and more: *umuDC* and the *Escherichia coli* SOS response.. Genetics.

[58] McAllister LJ, Tseng HJ, Ogunniyi AD, Jennings MP, McEwan AG (2004). Molecular analysis of the *psa* permease complex of *Streptococcus pneumoniae*.. Mol Microbiol.

[59] Brown JS, Gilliland SM, Holden DW (2001). A *Streptococcus pneumoniae* pathogenicity island encoding an ABC transporter involved in iron uptake and virulence.. Mol Microbiol.

[60] Berry AM, Paton JC (1996). Sequence heterogeneity of PsaA, a 37-kilodalton putative adhesin essential for virulence of *Streptococcus pneumoniae*.. Infect Immun.

[61] Brown JS, Gilliland SM, Ruiz-Albert J, Holden DW (2002). Characterization of *pit*, a *Streptococcus pneumoniae* iron uptake ABC transporter.. Infect Immun.

[62] Dintilhac A, Alloing G, Granadel C, Claverys JP (1997). Competence and virulence of *Streptococcus pneumoniae*: Adc and PsaA mutants exhibit a requirement for Zn and Mn resulting from inactivation of putative ABC metal permeases.. Mol Microbiol.

[63] Tseng HJ, McEwan AG, Paton JC, Jennings MP (2002). Virulence of *Streptococcus pneumoniae*: PsaA mutants are hypersensitive to oxidative stress.. Infect Immun.

[64] Busenlehner LS, Pennella MA, Giedroc DP (2003). The SmtB/ArsR family of metalloregulatory transcriptional repressors: Structural insights into prokaryotic metal resistance.. FEMS Microbiol Rev.

[65] Embry A, Hinojosa E, Orihuela CJ (2007). Regions of Diversity 8, 9 and 13 contribute to *Streptococcus pneumoniae* virulence.. BMC Microbiol.

[66] Quentin Y, Fichant G, Denizot F (1999). Inventory, assembly and analysis of *Bacillus subtilis* ABC transport systems.. J Mol Biol.

[67] Hava DL, Camilli A (2002). Large-scale identification of serotype 4 *Streptococcus pneumoniae* virulence factors.. Mol Microbiol.

[68] Lau GW, Haataja S, Lonetto M, Kensit SE, Marra A (2001). A functional genomic analysis of type 3 *Streptococcus pneumoniae* virulence.. Mol Microbiol.

[69] Paterson GK, Nieminen L, Jefferies JM, Mitchell TJ (2008). PclA, a pneumococcal collagen-like protein with selected strain distribution, contributes to adherence and invasion of host cells.. FEMS Microbiol Lett.

[70] Bagnoli F, Moschioni M, Donati C, Dimitrovska V, Ferlenghi I (2008). A second pilus type in *Streptococcus pneumoniae* is prevalent in emerging serotypes and mediates adhesion to host cells.. J Bacteriol.

[71] Avery OT, Macleod CM, McCarty M (1944). Studies on the Chemical Nature of the Substance Inducing Transformation of Pneumococcal Types : Induction of Transformation by a Desoxyribonucleic Acid Fraction Isolated from Pneumococcus Type Iii.. J Exp Med.

[72] Weiser JN, Austrian R, Sreenivasan PK, Masure HR (1994). Phase variation in pneumococcal opacity: relationship between colonial morphology and nasopharyngeal colonization.. Infect Immun.

[73] Horton RM, Ho SN, Pullen JK, Hunt HD, Cai Z (1993). Gene splicing by overlap extension.. Methods Enzymol.

[74] Morona JK, Miller DC, Morona R, Paton JC (2004). The effect that mutations in the conserved capsular polysaccharide biosynthesis genes *cpsA*, *cpsB*, and *cpsD* have on virulence of *Streptococcus pneumoniae*.. J Infect Dis.

[75] LeMessurier KS, Ogunniyi AD, Paton JC (2006). Differential expression of key pneumococcal virulence genes *in vivo*.. Microbiology.

[76] Ogunniyi AD, Giammarinaro P, Paton JC (2002). The genes encoding virulence-associated proteins and the capsule of *Streptococcus pneumoniae* are upregulated and differentially expressed *in vivo*.. Microbiology.

[77] Mahdi LK, Ogunniyi AD, LeMessurier KS, Paton JC (2008). Pneumococcal virulence gene expression and host cytokine profiles during pathogenesis of invasive disease.. Infect Immun.

[78] Livak KJ, Schmittgen TD (2001). Analysis of relative gene expression data using real-time quantitative PCR and the 2^−ΔΔCT^ Method.. Methods.

[79] Sambrook J, Fritsch EF, Maniatis T, Cold Spring Harbor Laboratory (1989). Molecular cloning : a laboratory manual.

[80] Carver TJ, Rutherford KM, Berriman M, Rajandream MA, Barrell BG (2005). ACT: the Artemis Comparison Tool.. Bioinformatics.

[81] Macrina FL, Evans RP, Tobian JA, Hartley DL, Clewell DB (1983). Novel shuttle plasmid vehicles for *Escherichia-Streptococcus* transgeneric cloning.. Gene.

[82] Giammarinaro P, Paton JC (2002). Role of RegM, a homologue of the catabolite repressor protein CcpA, in the virulence of *Streptococcus pneumoniae*.. Infect Immun.

[83] Martin B, Garcia P, Castanie MP, Glise B, Claverys JP (1995). The *recA* gene of *Streptococcus pneumoniae* is part of a competence-induced operon and controls an SOS regulon.. Dev Biol Stand.

