# Supplementary material for: A Variable Region within the Genome of Streptococcus pneumoniae Contributes to Strain-Strain Variation in Virulence
Source: PLoS One. 2011 May 5;6(5):e19650. doi: 10.1371/journal.pone.0019650 (PMC3088708; doi:10.1371/journal.pone.0019650)
Supplement: Table S1 — List of oligonucleotides used in this study. (DOC) [file pone.0019650.s001.doc]

**Table S1 Primers used in this study**

| **Confirmation of virulence-associated regions by PCR** | | |
| --- | --- | --- |
| **1** | **RH06Fa** | 5’- GGT CCT GGT CGT GAA CAA AC -3’ |
|  | **RH06R_(3)_** | 5’- GCC TCT CTC AAA GCC TCC CTC -3 |
|  | **RHrtPblBF** | 5’- GAA CGT TCA CAA CAG GAG GT -3’ |
|  | **RHrtPblBR** | 5’- TCA TTT ACT AGG GCG ACA GG -3’ |
| **2** | **RH224/5F** | 5’- GAG GAA GAG GAC ATA GAA AAT GG -3’ |
|  | **RH224/5R** | 5’- GCC CTC TGT ACC GGC TGG G -3’ |
| **3 & 4** | **a** | 5’- GTC CTA GCT CAG CGA GTA GAA G -3 |
|  | **c** | 5’- CGA ATA GAC CAC AAT CAA GCC C -3’ |
| **5** | **RH344F** | 5’- GTT GAA CCC GCA ATT CAG CCT G -3’ |
|  | **RH344R** | 5’- GAC ATG TGA CCA GGA AAC CAT TG -3’ |
|  | **RHzmpDF** | 5’- GAA CCG GAA GTT CAT GAG AA -3’ |
|  | **RHzmpDR** | 5’- CAC ATA CGG AAA GCA TTG TG -3’ |
|  | **RHumucF*** | 5’- CAT CGA TTG CGG ACT GGA GCC T -3’ |
|  | **RHumucR*** | 5’- CGC TTT GTG TTA TGA GTC GTG C -3’ |
|  | **RHint5252F*** | 5’- CCA TGC AAT GGA AAA ACC TC -3’ |
|  | **RHint5252R*** | 5’- CCA TGC AAT GGA AAA ACC TC -3’ |
|  | **RHint916F*** | 5’- GCG TGA TTG TAT CTC ACT -3’ |
|  | **RHint916R*** | 5’- GAC GCT CCT GTT GCT TCT -3’ |
| **6** | **RH383F** | 5’- CAA CAC GTC CAT AAT GAG CTG TAC -3’ |
|  | **RH383R** | 5’- CAA CAC GTC CAT AAT GAG CTG TAC -3’ |
| **7** | **RH467F** | 5’- GAT ACA TGG TCA ATA CCT CTA C -3’ |
|  | **RH467R** | 5’- CCA CTC CGT GAT ACC ATC C -3’ |
| **PI-2** | **1008for^+^** | 5’- GCT GGA TCG AGT TTG AAA CCA GAA -3’ |
|  | **1009rev^*^** | 5’- TAA GGA TCA CCA AAG TCC AAG GCA -3’ |
| **8** | **RH386F_(2)_** | 5’- YGA ATC CTG TGG AAC TAC TCC -3’ |
|  | **RH386R_(2)_** | 5’- GCC ATA TAG CAT TGT CCA TAA CG -3’ |
|  | **RH386F_(3)_** | 5’- GAA GGA GRT GAT AAA GTC CAT C -3’ |
|  | **RH386R_(3)_** | 5’- GGG GCA GTA TGG GAC TAC ATT TGG -3’  5’- GGG GAA GTA TGG GTC TAC ATT TGG -3’ |
| **Construction of D39ΔPezT** | | |
|  | **a** | 5’- GTC CTA GCT CAG CGA GTA GAA G -3 |
|  | **aq** | 5’- GTC ACG ATT CTG TTT GTA GAA CC -3’ |
|  | **J214** | 5’- GAA GGA GTG ATT ACA TGA ACA A -3’ |
|  | **J215** | 5’- CTC ATA GAA TTA TTT CCT CCC G -3’ |
|  | **ec** | 5’- CGG GAG GAA ATA ATT CTA TGA GGT TCA AAA TGT CCA GTT TGG ACA -3’ |
|  | **g** | 5’- CCT GAT AAT CTT CCT GCG TTG -3’ |
| **Construction of D39ΔPPI-1** | | |
|  | **t** | 5’- GAG AGA TTA TCG ATG GTT ATA TTG G -3’ |
|  | **ed** | 5’- GAG ATC CGG CCG ATG AAG ATT TTC TAG AGA ATT TTC -3’ |
|  | **RHcatF** | 5’- TAT AAT CGG CCG CAA CAG CGT GAC CGA AAA TTG -3’ |
|  | **RHcatR** | 5’- TAT AAT CTC GAG GGG TTC CGA GGC TCA ACG TC -3’ |
|  | **ee** | 5’- ATA TAT CTC GAG GTT CAA AAT GTC CAG TTT GGA CA -3’ |
| **Construction of D39^1861^** | | |
|  | **ef** | 5’- CAA CAA TCT TAC CAC CTT TCA TT -3’ |
|  | **eg** | 5’- CGT TTT GAA CGT GGG GAG TC -3’ |
|  | **J293a** | 5’- GAT CAT CGG CCG GGT TCG CGG GAA GTC TAC TAA G -3’ |
|  | **J254a** | 5’- TGC ATA CTC GAG TTA TAC CTT CTT CAA TCT GTT ATT TAA ATA GTT TAT AGT TA -3’ |
|  | **eh** | 5’- TGC ATA CTC GAG GTT GGT GGT TAA ATC ACT TAG GTG -3’ |
|  | **c** | 5’- CGA ATA GAC CAC AAT CAA GCC C -3’ |
| **Construction of D39^1^** | | |
|  | **af** | 5’- GTC AGA TGG AGT TAA CGG ATG G -3’ |
|  | **ei** | 5’- CGA ACA GTT GAT GTT CCA AAG A -3’ |
|  | **ej** | 5’- CAA GTG GGC TAT ATG GAA CC -3’ |
| **Real time RT-PCR** | | |
| **16S** | **RH16SF_(3)_** | 5’- CAT GCA AGT AGA ACG CTG AA -3’ |
|  | **RH16SR_(3)_** | 5’- TGT CAT GCA ACA TCC ACT CT -3’ |
| ***pblB*** | **RHrtPblBF** | 5’- GAA CGT TCA CAA CAG GAG GT -3’ |
|  | **RHrtPblBR** | 5’- TCA TTT ACT AGG GCG ACA GG -3’ |
| **Endolysin** | **RH0083F** | 5’- TGG CTC AGG CTA TAT GCT TT -3’ |
|  | **RH0083R** | 5’- TCA TGG CAC CTT CTA CAT CA -3’ |
| **Na^+^ dep. transporter** | **RH0747F** | 5’- TCC AGT CAG GAA TCT CCA TC -3’ |
|  | **RH0747R** | 5’- AAA GAG TTG AGG CAA GAC GA -3’ |
| ***nplT*** | **RH6F** | 5’- ATA CAG AGC GAA TCC TGT GG -3’ |
|  | **RH6R** | 5’- GAC GAC AAT CTG GAT CTG GT -3’ |
| ***pezAT*** | **RH10F** | 5’- AAA GTT GTA GCC ATG CTT GA -3’ |
|  | **RH10R** | 5’- TGA CGT TGA GCT TTC ACA TC -3’ |
| **3HIBDH** | **RH16F** | 5’- CGG AAC CAA CAA AAT TCA AG -3’ |
|  | **RH16R** | 5’- CAT CCA ACC ATC ATT ACA TAC G -3’ |
| **Biotin carboxylase** | **RH22F** | 5’- AAA GCA TTG GAA GAA TGT CG -3’ |
|  | **RH22R** | 5’- AAT ATC TCC CCC TCC AAA TC -3’ |
| **Major facilitator** | **RH23F** | 5’- CAG ATA GTT TCA AGC GGA AAA -3’ |
|  | **RH23R** | 5’- GCC CTG AAA AAG CAC TCA TA -3’ |
| **Fe^2+^/Pb^2+^ permease** | **RH1340F** | 5’- GTT GAC CTA GAT GCG GAA AA -3’ |
|  | **RH1340R** | 5’- CAC GAA CCA CAC TTT CAT TG -3’ |
| **Gly. Hydrolase** | **RH1353F** | 5’- CGT CCA GAA TAT CCA GGT GT -3’ |
|  | **RH1353R** | 5’- TTC CTT CGA TTG CGT AGA TT -3’ |

*Primers reported in Henderson-Begg *et al*., 2009

^+^Primers reported in Bagnolis *et al*., 2008
